# The predictive value of the pre-PCI prognostic nutritional index combined with the geriatric nutritional risk index for one-year outcomes in patients with chronic total occlusion

**DOI:** 10.3389/fnut.2025.1707981

**Published:** 2025-11-26

**Authors:** Zhang Bin, Sun YuRong, Bai Hangrui, Zhang JingSi, Lu Yi, Liu FengYi, Yang Qian, Zhang YangYou, Luan Bo, Ding YanChun, Zhang XiaoJiao

**Affiliations:** 1 Department of Cardiology, The People’s Hospital of Liaoning Province, The People’s Hospital of China Medical University, Shenyang, Liaoning, China; 2 Department of Cardiology, The Second Hospital of DaLian Medical University, Dalian, Liaoning, China

**Keywords:** prognostic nutritional index, geriatric nutritional risk index, chronic total occlusion, percutaneous coronary intervention, short-term outcomes

## Abstract

**Aim:**

Chronic total occlusion (CTO) is associated with high rates of major adverse cardiovascular and cerebrovascular events (MACCEs) after percutaneous coronary intervention (PCI). Nutritional and inflammatory status are increasingly recognized as key prognostic factors. This study aimed to evaluate the predictive value of the prognostic nutritional index (PNI) combined with the geriatric nutritional risk index (GNRI) for MACCEs in CTO patients undergoing PCI.

**Methods:**

A total of 395 CTO patients from Northeast China who were treated with PCI at Liaoning Provincial People’s Hospital between February 2019 and December 2023 were retrospectively analyzed. Baseline clinical, laboratory, and procedural data were collected. The PNI and GNRI scores were calculated based on pre-PCI laboratory test results, and patients were followed for 12 months to monitor the occurrence of MACCEs. Independent predictors were identified using logistic regression analysis, and the predictive performance of three models was evaluated using ROC curves, C-statistics, net reclassification improvement (NRI), integrated discrimination improvement (IDI), and Kaplan–Meier survival analysis.

**Results:**

During follow-up, 125 patients (31.6%) experienced MACCEs. Both PNI-GNRI were independent predictors of MACCEs risk. Adding PNI to the baseline risk model increased the C-statistic from 0.696 to 0.770 (*p* < 0.001). Incorporating GNRI further increased it to 0.826 (*p* < 0.001), with significant improvements in NRI (0.308) and IDI (0.207). Kaplan–Meier analysis demonstrated that patients with low PNI or GNRI scores had significantly higher cumulative incidence of MACCEs. Subgroup analyses confirmed the stability of these associations across various patient strata.

**Conclusion:**

Both PNI-GNRI are independent predictors of MACCEs, and their combined model provides superior prognostic stratification for CTO patients compared with traditional risk models, particularly in elderly patients. Comprehensive assessment of nutritional and inflammatory status enables precise perioperative risk stratification. It also offers guidance for individualized management, nutritional interventions, and long-term rehabilitation.

## 1 Introduction

Coronary heart disease (CHD) is a common cardiovascular disorder caused by myocardial ischemia, hypoxia, and/or necrosis. Its primary pathological mechanism is the development of coronary atherosclerosis, leading to vascular narrowing or even complete occlusion. As one of the most prevalent types of cardiovascular disease, CHD affects millions of people worldwide (1, 2).

Although the mortality of coronary artery disease (CAD) has declined in recent decades, it remains a leading cause of death worldwide and imposes a major public health and economic burden (3). Epidemiological data indicate that CHD accounts for approximately 32.7% of global heart disease and 1.7% of the total disease burden (4). Chronic total occlusion (CTO) is detected in 15–30% of patients undergoing coronary angiography and is characterized by occlusion lasting over 3 months with insufficient collateral circulation, leading to persistent ischemia and angina (5). Compared with non-CTO lesions, CTO is associated with lower success rates of percutaneous coronary intervention (PCI) and higher risks of adverse cardiovascular and cerebrovascular events (6). In recent years, growing evidence has demonstrated that nutritional imbalance is closely associated with poor clinical outcomes in patients with cardiovascular diseases (7, 8). Adequate nutrition not only helps maintain a good quality of life but also reduces the risk of chronic diseases (9). Therefore, systematic nutritional screening and intervention in patients with cardiovascular diseases are considered key strategies for preventing cachexia (10).

The objective quantification of nutritional status has long been a challenge in clinical research. The prognostic nutritional index (PNI), one of the commonly used tools, is calculated based on serum albumin concentration and lymphocyte count, thereby providing a comprehensive assessment of nutritional and immune status. Originally developed for nutritional evaluation in patients undergoing gastrointestinal surgery, PNI has increasingly been investigated for prognostic prediction in patients with CAD. Current evidence suggests that malnutrition not only independently predicts mortality and major adverse cardiovascular and cerebrovascular events (MACCEs) in CAD patients (11), but that a low PNI value is also closely associated with the presence of multivessel coronary lesions (12). However, the effectiveness of PNI in predicting clinical outcomes remains inconsistent across studies, and available evidence is still limited (13). Another nutritional assessment indicator is the geriatric nutritional risk index (GNRI), proposed by Bouillanne et al. (14), which is calculated using serum albumin levels and the ratio of actual to ideal body weight. GNRI is a simple and reliable scoring method for assessing nutritional risk. It can predict morbidity and mortality in elderly patients and has been widely used to evaluate the association between nutritional status and adverse outcomes in diverse populations. Previous studies have demonstrated that malnutrition identified by GNRI at admission is an independent predictor of MACCEs in patients with CTO undergoing PCI (6, 15). Nevertheless, evidence regarding the relationship between malnutrition and adverse cardiovascular and cerebrovascular events in CTO patients after PCI remains scarce.

Therefore, the present study aims to evaluate the predictive value of GNRI combined with PNI for MACCEs in CTO patients treated with PCI. The goal is to provide new perspectives and evidence-based insights for risk stratification and prognosis management in this population.

## 2 Methods

### 2.1 Patient population

**Figure 1:**
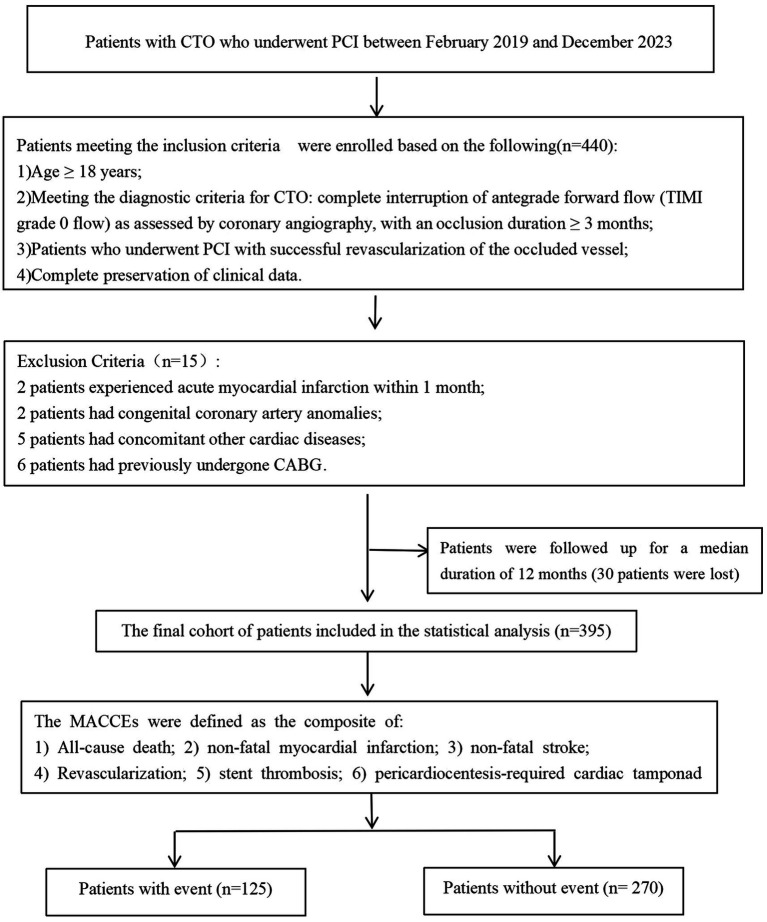
Flow diagram of participant selection.

From February 2019 to December 2023, a total of 440 patients who were hospitalized at Liaoning Provincial People’s Hospital for the first time and diagnosed with at least one CTO in a major coronary artery (left anterior descending artery, left circumflex artery, or right coronary artery) by coronary angiography were screened. According to the exclusion criteria, 15 patients (3.4%) were excluded, and 30 patients (6.8%) were lost to follow-up. Ultimately, 395 patients were included in this study (Figure 1). As a retrospective analysis, it was approved by the Ethics Committee of Liaoning Provincial People’s Hospital (Approval No.2024-K063).

### 2.2 Inclusion criteria

(1) Age ≥18 years; (2) Meeting the diagnostic criteria for CTO: complete interruption of antegrade flow (TIMI grade 0) on coronary angiography with an occlusion duration of ≥3 months; (3) Undergoing PCI with successful revascularization of the occluded vessel; (4) Availability of complete clinical data.

### 2.3 Exclusion criteria

(1) Contraindications to PCI or contrast agent administration; (2) History of acute myocardial infarction within 1 month, or emergency coronary angiography/stent implantation during the acute phase; (3) Congenital coronary artery anomalies; (4) Connective tissue diseases involving the coronary arteries; (5) Other coexisting cardiac diseases, such as congenital heart disease, severe valvular disease, cardiomyopathy, or cor pulmonale; (6) Previous intravenous thrombolysis, thrombectomy, or coronary artery bypass grafting (CABG); (7) Severe hepatic or renal dysfunction; (8) Severe infectious diseases; (9) Presence of malignancy or systemic immunodeficiency disorders; (10) Patients with nephrotic syndrome, hypersensitivity reactions, hematologic malignancies, systemic lupus erythematosus, or other diseases known to significantly affect albumin or lymphocyte levels.

### 2.4 Follow-up

All patients were followed up at 6 and 12 months after PCI, through multiple approaches including but not limited to telephone interviews and outpatient visits. Information on adverse events was obtained either by telephone contact with patients or their family members, or during outpatient follow-up visits. During the follow-up, 30 patients (6.8%) were lost, and a total of 395 patients successfully completed follow-up.

### 2.5 Study endpoints

Based on the occurrence of MACCEs within 12 months after PCI, patients were divided into two groups: the MACCEs group and the non-MACCEs group. Demographic characteristics, laboratory parameters, imaging findings, coronary angiography results, GNRI scores, and PNI scores were compared between the two groups.

### 2.6 Definitions

GNRI = [1.489 × albumin (g/L)] + [41.7 × (weight/ideal body weight WLo)].

Ideal body weight (WLo) was calculated using the Lorentz formula:

Male: H-100-[(H-150)/4]; Female: H-100-[(H-150)/2.5] (14).

PNI = 10 × serum albumin (g/L) + 5 × total lymphocyte count (10^9^/L) (16).

### 2.7 Statistical analysis

All statistical analyses were conducted using SPSS version 26.0 and R version 4.3.2. Continuous variables were presented as mean ± standard deviation, and categorical variables as counts and percentages. Between-group comparisons were performed using the independent-samples *t*-test or the non-parametric rank-sum test for continuous variables, and the chi-square test for categorical variables. Univariate logistic regression was initially employed to identify potential predictors of MACCEs. Variables with *p* < 0.05 were subsequently entered into a multivariate logistic regression model, with results expressed as odds ratios (ORs) and 95% confidence intervals (CIs). Model discrimination was assessed by receiver operating characteristic (ROC) curve analysis and the C-statistic, while subgroup effect sizes were illustrated using forest plots. Furthermore, the net reclassification improvement (NRI) and integrated discrimination improvement (IDI) were calculated to evaluate the incremental predictive value and clinical utility of the novel model. Survival curves were generated with the Kaplan–Meier method and compared using the log-rank test. A two-sided *p* < 0.05 was considered statistically significant.

## 3 Results

### 3.1 Baseline characteristics of the study population

A total of 395 patients meeting the inclusion criteria were enrolled, with a median follow-up of 12 months. Among them, 125 patients (31.65%) experienced MACCEs, while 270 patients (68.35%) did not. The baseline characteristics of the overall cohort are summarized in Table 1. Compared with the non-MACCEs group, patients in the MACCEs group showed statistically significant differences in the prevalence of diabetes, hypertension, and current smoking, as well as in lymphocyte count, serum albumin, low-density lipoprotein cholesterol (LDL-C), GNRI score, PNI score, the number of CTO-involved vessels, number of stents implanted, and total stent length (all *p* < 0.05). Overall, patients in the MACCEs group exhibited a higher prevalence of cardiovascular metabolic and lifestyle-related risk factors, particularly diabetes, hypertension, and smoking, compared with those without MACCEs (all p < 0.05). In addition, these patients demonstrated poorer lipid control, as evidenced by elevated LDL-C levels, indicating a greater burden of dyslipidemia. With respect to nutritional and inflammatory parameters, the MACCEs group showed significantly higher lymphocyte counts but lower GNRI and PNI scores, as well as reduced serum albumin levels, suggesting a state of impaired nutritional status and systemic inflammatory response. Regarding PCI-related characteristics, patients who experienced MACCEs had a greater number of CTO-involved vessels, received more stents, and had longer total stent lengths than those in the non-MACCEs group (all *p* < 0.05).

**Table 1:** Baseline clinical characteristics.

Variable	All subjects (*n* = 395)	MACCEs (*n* = 125)	non-MACCEs (*n* = 270)	*P*-value
Age, years	61.85 ± 10.57	60.91 ± 10.43	62.29 ± 10.62	0.230
Man, *n* (%)	296(74.9)	94(75.2)	202(74.8)	0.935
BMI	25.47 ± 3.35	25.53 ± 3.55	25.45 ± 3.27	0.827
Diabetes mellitus, *n* (%)	194(49.1)	73(58.4)	121(44.8)	0.012
Hypertension, *n* (%)	255(64.6)	94(75.2)	161(59.6)	0.003
Current smoking	156(39.5)	60(48.0)	96(35.6)	0.019
LVEF (%)	44.91 ± 7.14	45.28 ± 6.96	44.74 ± 7.23	0.486
TC (mmol/L)	3.86 ± 1.06	3.93 ± 1.01	3.83 ± 1.09	0.363
TG (mmol/L)	1.72 ± 1.11	1.73 ± 0.88	1.72 ± 1.20	0.944
LDL-C (mmol/L)	2.28 ± 0.86	2.55 ± 0.86	2.15 ± 0.83	<0.001
HDL-C (mmol/L)	0.98 ± 0.33	0.96 ± 0.27	0.98 ± 0.35	0.609
Hb (g/L)	137.87 ± 17.15	138.46 ± 15.40	137.59 ± 17.93	0.639
WBC (10^9^/L)	7.23 ± 2.17	7.45 ± 2.47	7.13 ± 2.01	0.165
NEU (10^9^/L)	4.76 ± 1.95	4.97 ± 2.43	4.67 ± 1.70	0.185
LYM (10^9^/L)	1.82 ± 0.62	1.95 ± 0.61	1.76 ± 0.61	0.005
MON (10^9^/L)	1.12 ± 1.95	1.01 ± 1.67	1.17 ± 2.07	0.478
PLT (10^9^/L)	220.46 ± 60.38	226.18 ± 57.64	217.81 ± 61.53	0.200
ALT (U/L)	28.60 ± 22.83	29.82 ± 29.57	28.03 ± 18.95	0.469
AST (U/L)	25.01 ± 17.71	26.30 ± 21.63	24.40 ± 15.57	0.323
HbA1c (%)	6.60 ± 1.58	6.62 ± 1.62	6.59 ± 1.57	0.892
FPG (mmol/L)	6.70 ± 2.51	6.67 ± 2.42	6.72 ± 2.55	0.861
Cr (μmol/L)	77.62 ± 75.40	85.69 ± 18.66	73.92 ± 90.13	0.149
BUN (mmol/L)	6.34 ± 2.46	6.28 ± 2.14	6.36 ± 2.60	0.760
Alb (g/L)	42.23 ± 5.22	38.86 ± 4.27	43.78 ± 4.89	<0.001
GNRI	104.09 ± 7.87	99.02 ± 6.54	106.44 ± 7.31	<0.001
PNI	51.33 ± 5.89	48.61 ± 4.76	52.60 ± 5.94	<0.001
Number of CTO arteries	1.12 ± 0.34	1.20 ± 0.42	1.09 ± 0.29	0.002
J-CTO score	2.77 ± 0.79	2.68 ± 0.76	2.81 ± 0.81	0.127
Number of stents implanted	2.41 ± 1.12	2.60 ± 1.22	2.32 ± 1.05	0.021
Overall stent length (mm)	68.78 ± 32.13	74.34 ± 35.78	66.20 ± 30.01	0.019
Total procedural time (min)	93.51 ± 49.59	96.66 ± 54.35	92.04 ± 47.26	0.390
Contrast volume (mL)	234.80 ± 100.54	248.28 ± 110.38	228.56 ± 95.21	0.070

p-values < 0.05. BMI, Body Mass Index; LVEF, left ventricular ejection fraction; TC, total cholesterol; TG, triacylglycerol; LDL-C, low-density lipoprotein cholesterol; HB, hemoglobin; WBC, white blood cell; PLT, platelet; HDL-C, high-density lipoprotein cholesterol; NEU, neutrophil; MON, monocyte; LYM, lymphocyte; AST, aspartate aminotransferase; Cr, creatinine; ALT, alanine aminotransferase; FPG, fasting plasma glucose; Alb, albumin; GNRI, geriatric nutritional risk index; PNI, prognostic nutritional index; MACCEs, major adverse cardiac and cerebrovascular events.

### 3.2 Risk factor analysis

Univariate and multivariate logistic regression analyses were performed to identify predictors of MACCEs in this cohort (Table 2). The results demonstrated that diabetes, hypertension, current smoking, LDL-C, GNRI score, PNI score, number of CTO-involved vessels, number of stents implanted, and total stent length were all significant risk factors for MACCEs (all *p* < 0.05). Detailed findings are as follows: A history of diabetes was significantly associated with an increased risk of the primary endpoint in both univariate (OR = 1.729, 95%CI: 1.126–2.655, *p* = 0.012) and multivariate analyses (OR = 2.029, 95%CI: 1.199–3.433, *p* = 0.008). Hypertension was also strongly correlated with an increased risk of MACCEs in univariate (OR = 2.053, 95%CI: 1.279–3.295, *p* = 0.003) and multivariate analyses (OR = 2.151, 95%CI: 1.217–3.803, *p* = 0.008). Elevated LDL-C was significantly associated with MACCEs in univariate (OR = 1.721, 95%CI: 1.332–2.224, *p* < 0.001) and multivariate analyses (OR = 1.594, 95%CI: 1.177–2.159, *p* = 0.003). Lower GNRI scores were strongly predictive of MACCEs in univariate (OR = 0.852, 95%CI: 0.819–0.887, *p* < 0.001) and multivariate analyses (OR = 0.802, 95%CI: 0.749–0.860, *p* < 0.001). Similarly, PNI was significantly associated with the primary endpoint, as shown in univariate (OR = 0.872, 95%CI: 0.833–0.912, *p* < 0.001) and multivariate analyses (OR = 1.091, 95%CI: 1.005–1.185, *p* = 0.037). Although the adjusted OR for PNI in the multivariate analysis is slightly above 1, this may reflect the influence of multicollinearity and adjustment for confounding factors. The overall results from the univariate analysis, as well as subsequent ROC and Kaplan–Meier survival analyses, consistently indicate that higher PNI is associated with better outcomes. Moreover, the number of CTO-involved vessels was significantly correlated with MACCEs occurrence in univariate (OR = 2.472, 95%CI: 1.368–4.470, *p* = 0.003) and multivariate analyses (OR = 2.362, 95%CI: 1.101–5.068, *p* = 0.027).

**Table 2:** Univariate and multivariate logistic regression analysis.

Variable	Univariate analysis		Multivariate analysis	
	OR (95% CI)	*P*-value	OR (95% CI)	*P*-value
Age	0.988(0.968–1.008)	0.230		
Man	1.021(0.625–1.667)	0.935		
BMI	1.007(0.945–1.073)	0.826		
Diabetes mellitus	1.729(1.126–2.655)	0.012	2.029(1.199–3.433)	0.008
Hypertension	2.053(1.279–3.295)	0.003	2.151(1.217–3.803)	0.008
Current smoking	1.673(1.088–2.573)	0.019	1.619(0.953–2.751)	0.075
LVEF	1.011(0.981–1.041)	0.485		
LDL-C	1.721(1.332–2.224)	<0.001	1.594(1.177–2.159)	0.003
Hb	1.003(0.991–1.016)	0.638		
WBC	1.070(0.972–1.177)	0.167		
PLT	1.002(0.999–1.006)	0.201		
ALT	1.003(0.994–1.012)	0.472		
AST	1.006(0.994–1.017)	0.333		
HbA1c	1.009(0.883–1.153)	0.891		
FPG	0.992(0.991–1.081)	0.861		
Cr	1.002(0.999–1.005)	0.182		
GNRI	0.852(0.819–0.887)	<0.001	0.802(0.749–0.860)	<0.001
PNI	0.872(0.833–0.912)	<0.001	1.091(1.005–1.185)	0.037
Number of CTO arteries	2.472(1.368–4.470)	0.003	2.362(1.101–5.068)	0.027
J-CTO score	0.810(0.618–1.062)	0.127		
Number of stents implanted	1.246(1.032–1.504)	0.022	1.138(0.589–2.200)	0.700
Overall stent length	1.008(1.001–1.015)	0.020	1.000(0.978–1.023)	0.969
Total procedural time	1.002(0.998–1.006)	0.389		
Contrast volume	1.002(1.000–1.004)	0.072		

OR, odds ratio; CI, confidence interval. *P*-values < 0.05.

BMI, Body Mass Index; LVEF, left ventricular ejection fraction; HB, hemoglobin; LDL-C, low-density lipoprotein cholesterol; HB, hemoglobin; PLT, platelet; ALT, alanine aminotransferase; AST, aspartate aminotransferase; Cr, creatinine; FPG, fasting plasma glucose; PNI, prognostic nutritional index; GNRI, geriatric nutritional risk index.

### 3.3 Evaluation of PNI-GNRI in MACCEs risk prediction models

**Figure 2:**
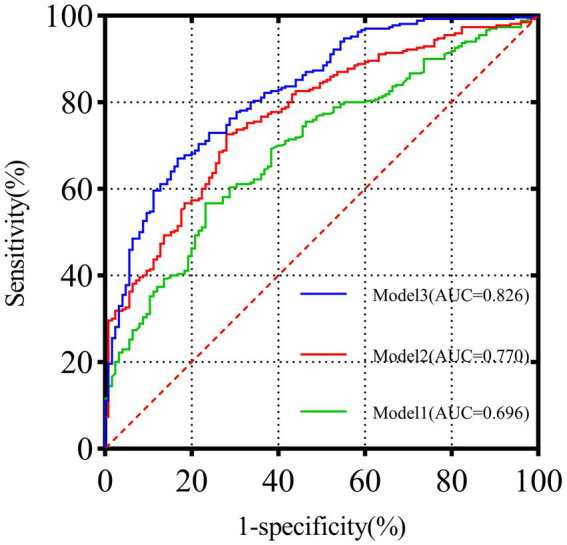
ROC curve analysis of the three models.

The synergistic effect of combining the PNI-GNRI in predicting MACCEs after PCI in CTO patients is shown in Table 3 and Figure 2. Compared with the baseline model including established risk factors (Model 1), the addition of PNI (Model 2) improved the C-statistic from 0.696 [95% CI (0.643–0.750), *p* < 0.001] to 0.770 [95% CI (0.723–0.818), *p* < 0.001]. Furthermore, incorporating both PNI-GNRI (Model 3) significantly increased the C-statistic from 0.696 [95% CI (0.643–0.750), *p* < 0.001] to 0.826 [95% CI (0.783–0.868), *p* < 0.001], demonstrating the strongest incremental effect. In addition, this combined model markedly improved the NRI by 0.308 [95% CI (0.239–0.376), *p* < 0.001] and the IDI by 0.207 [95% CI (0.163–0.251), *p* < 0.001]. A higher NRI indicated that incorporating nutritional indices improved the accuracy of patient risk reclassification for MACCEs, while an increased IDI reflected enhanced overall discrimination and predictive performance of the model. These results demonstrate that adding nutritional parameters meaningfully strengthened the model’s clinical predictive value.

**Table 3:** Evaluation of predictive models for MACCEs.

Variables	NRI		IDI		C-statistics	
	Index (95%CI)	*P*-value	Index (95%CI)	*P*-value	Index (95%CI)	*P*-value
Model 1		ref		ref	0.696 (0.643–0.750)	<0.001
Model 2	0.199 (0.147–0.251)	<0.001	0.090 (0.061–0.119)	<0.001	0.770 (0.723–0.818)	<0.001
Model 3	0.308 (0.0.239–0.376)	<0.001	0.207 (0.163–0.251)	<0.001	0.826 (0.783–0.868)	<0.001

Model 1: Diabetes mellitus + Hypertension + Number of CTO arteries + LDL-C.

Model 2: Diabetes mellitus + Hypertension + Number of CTO arteries + LDL-C + PNI.

Model 3: Diabetes mellitus + Hypertension + Number of CTO arteries + LDL-C + PNI + GNRI.

### 3.4 Subgroup analyses

**Figure 3:**
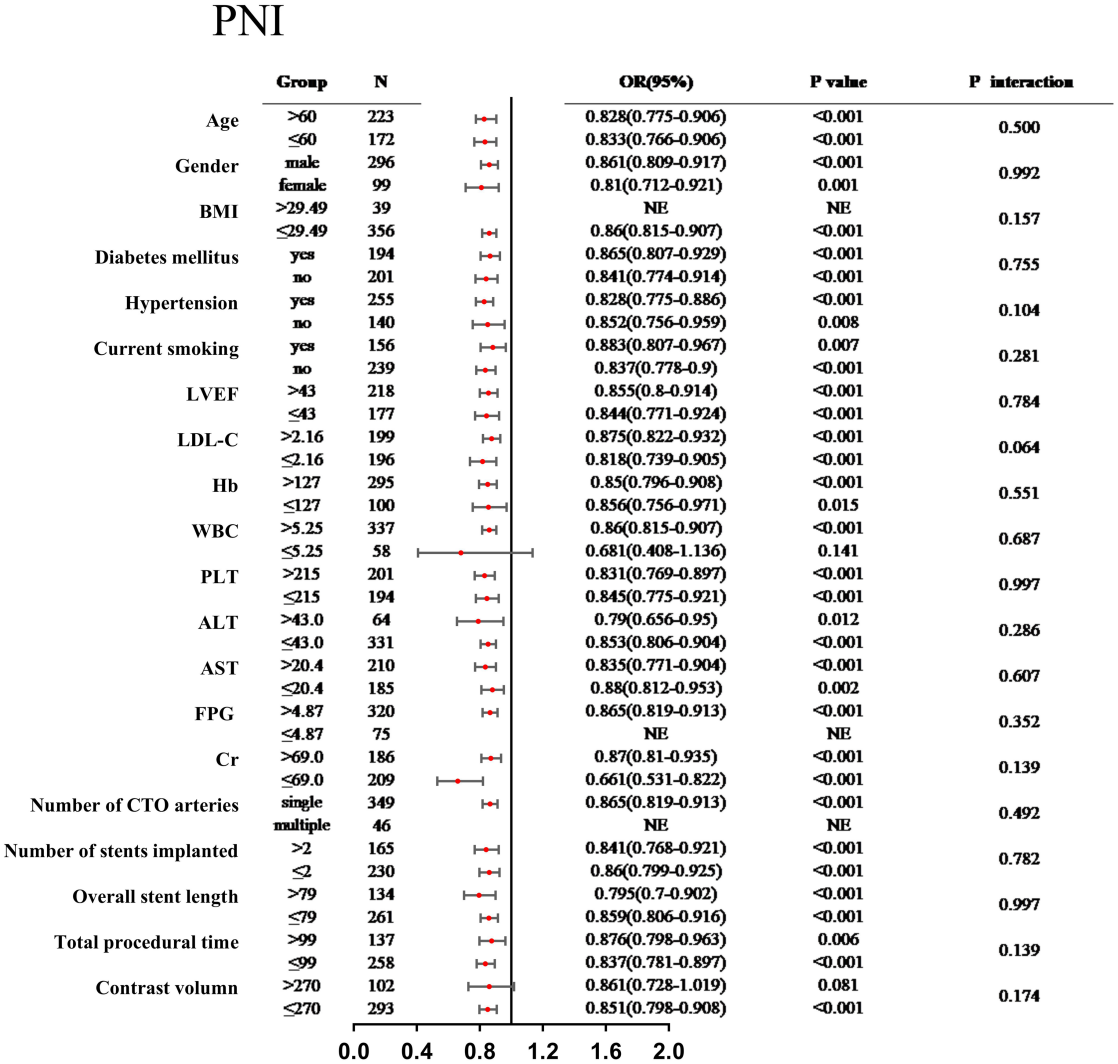
Subgroup analysis between the PNI and MACCEs across various subgroups.

**Figure 4:**
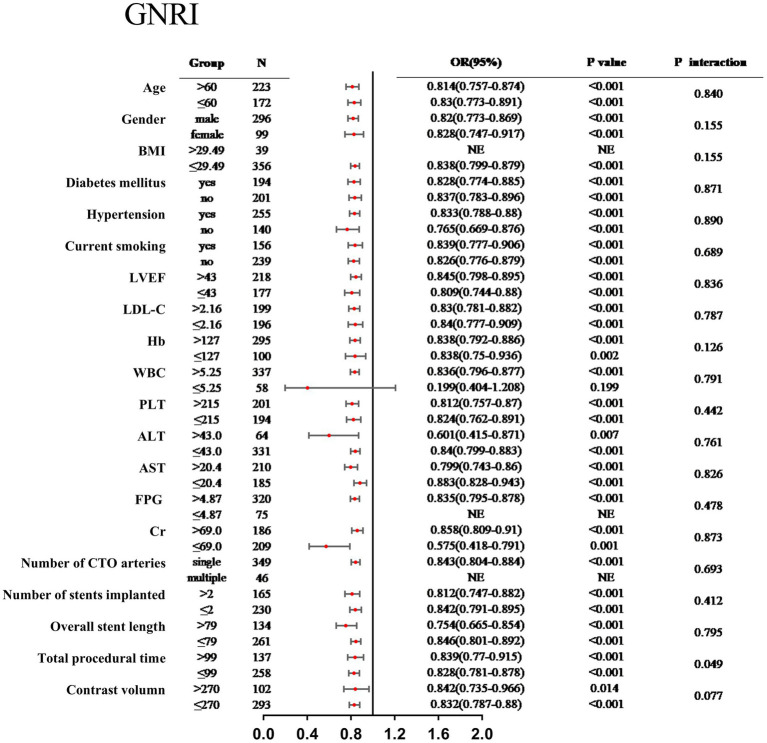
Subgroup analysis between the GRNI and MACCEs across various subgroups.

Subgroup analyses demonstrated that the relationship between the PNI and the risk of MACCEs remained consistent across various patient strata, including age, sex, BMI, diabetes mellitus, hypertension, current smoking status, LVEF, LDL-C, Hb, WBC, PLT, ALT, AST, FPG, Cr, number of CTO vessels, number of stents implanted, overall stent length, total procedural time, and contrast volume, with no significant interactions observed (all *P* for interaction > 0.05) (Figure 3). Subgroup analyses demonstrated that the relationship between the GNRI and the risk of MACCEs remained consistent across various patient strata, including age, sex, BMI, diabetes mellitus, hypertension, current smoking status, LVEF, LDL-C, Hb, WBC, PLT, ALT, AST, FPG, Cr, number of CTO vessels, number of stents implanted, overall stent length, and contrast volume, with no significant interactions observed (all *P* for interaction > 0.05) (Figure 4). Notably, a significant interaction was observed between GNRI and procedural time (*P* for interaction = 0.049), suggesting that procedural time may modify the prognostic impact of GNRI.

### 3.5 Predictive value of the PNI-GNRI for the risk of MACCEs

**Figure 5:**
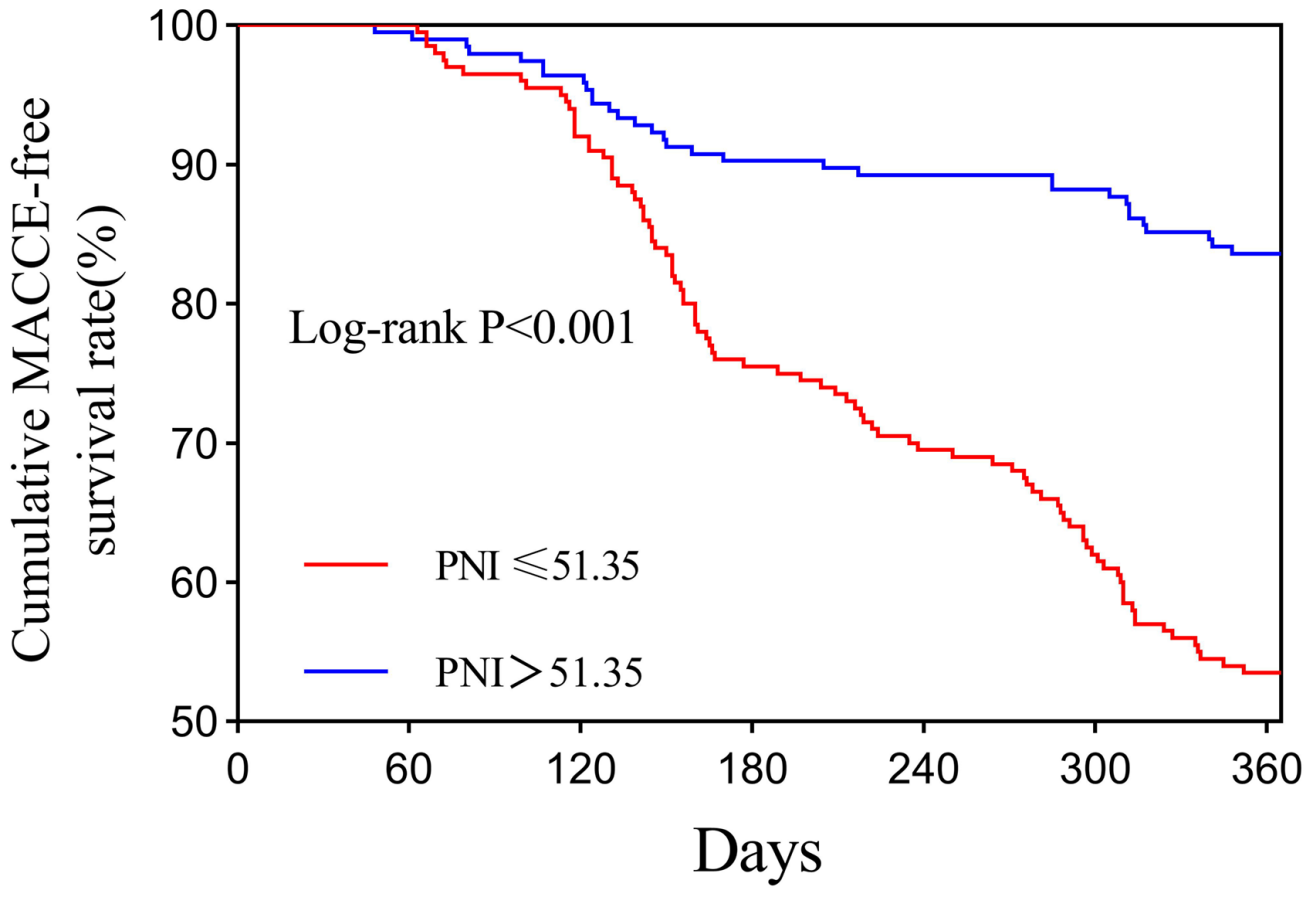
The PNI and risk: Kaplan–Meier curves for the incidences of MACCEs. The optimal cut-off value of PNI (≤51.35) was determined using ROC curve analysis by maximizing the Youden index (sensitivity + specificity − 1).

**Figure 6:**
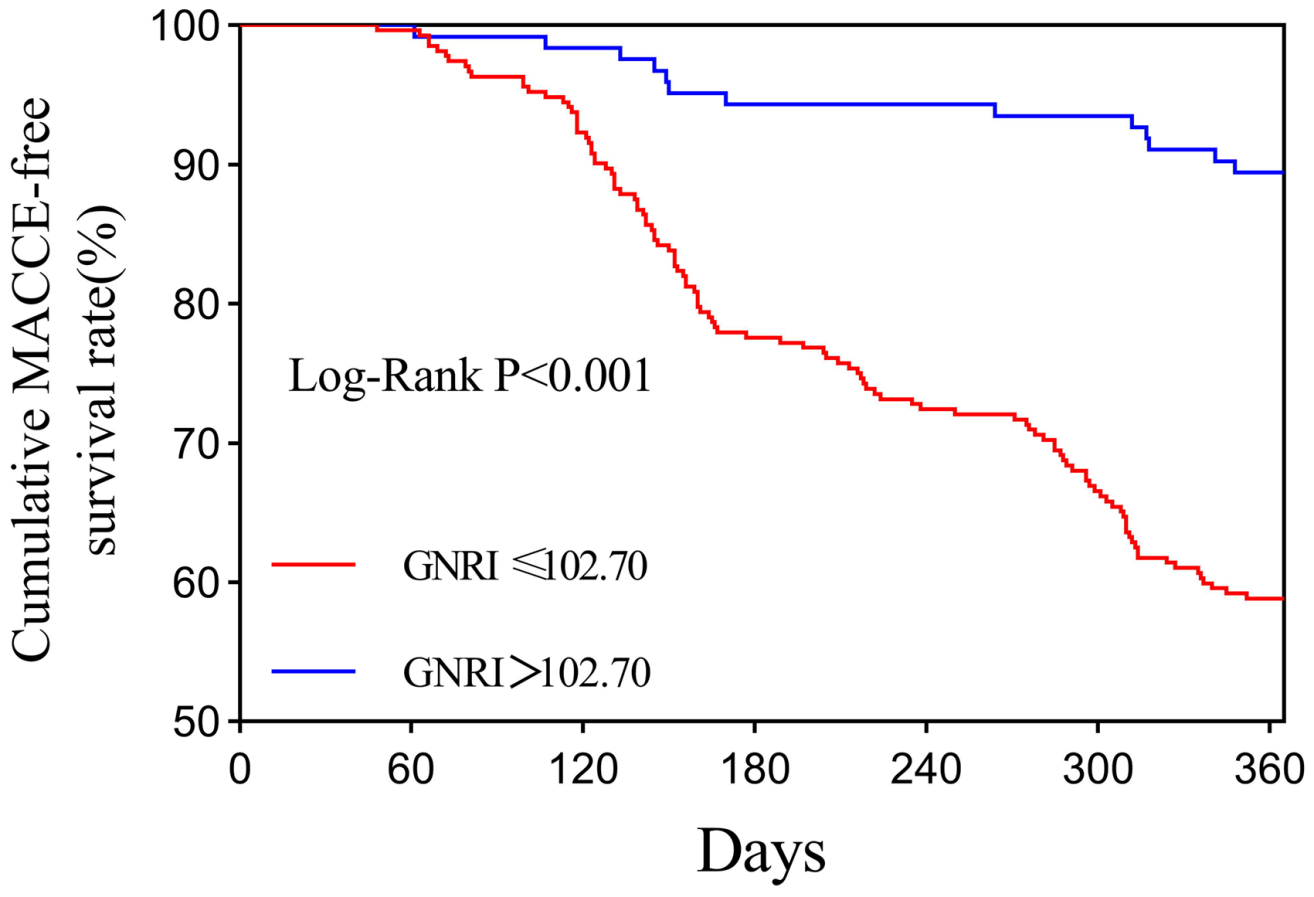
The PNI and risk: Kaplan–Meier curves for the incidences of MACCEs. The optimal cut-off value of GNRI (≤102.70) was determined using ROC curve analysis by maximizing the Youden index (sensitivity + specificity − 1).

The Kaplan–Meier analysis demonstrated that patients with a low PNI (≤51.35) exhibited a significantly higher cumulative incidence of MACCEs compared with those with higher PNI levels (log-rank test, *p* < 0.001) (Table 4 and Figure 5). Similarly, patients with a low GNRI (≤102.70) experienced a markedly increased cumulative incidence of MACCEs following PCI (log-rank test, *p* < 0.001) (Table 4 and Figure 6). Overall, the risk of MACCEs was substantially elevated in both the low PNI and low GNRI groups. The incidence of MACCEs in the high PNI group was reduced by 47.1% compared with the low PNI group (HR = 0.529, 95% CI: 0.330–0.847, *p* = 0.008). Similarly, patients in the high GNRI group experienced a 65.4% reduction in MACCEs incidence compared with the low GNRI group (HR = 0.346, 95% CI: 0.177–0.676, *p* = 0.002).

**Table 4:** Clinical outcomes on follow-up after one-year.

PNI				
Variable	All subjects (*n* = 395)	PNI > 51.35 (*n* = 195)	PNI ≤ 51.35 (*n* = 200)	*P*-value
MACCEs Incidence Rate	125(31.6%)	32(16.4%)	93(46.5%)	<0.001
Cardiac mortality	28(7.1%)	5(2.6%)	23(11.5%)	0.001
Myocardial infarction	86(21.8%)	24(12.3%)	62(31.0%)	<0.001
Target vessel revascularization	90(22.8%)	26(13.3%)	64(32.0%)	<0.001
In-stent thrombosis	71(18%)	21(10.8%)	50(25.0%)	<0.001
Nonfatal stroke	21(5.3%)	8(4.1%)	13(6.5%)	0.288

GNRI				
Variable	All subjects (*n* = 395)	GNRI > 107.7 (*n* = 123)	GNRI ≤ 107.7 (*n* = 272)	*P*-value
MACCEs Incidence Rate	125(31.6%)	13(10.6%)	112(41.2%)	<0.001
Cardiac mortality	28(7.1%)	2(1.6%)	26(9.6%)	0.004
Myocardial infarction	86(21.8%)	10(8.1%)	76(27.9%)	<0.001
Target vessel revascularization	90(22.8%)	11(8.9%)	79(29.0%)	<0.001
In-stent thrombosis	71(18%)	8(6.5%)	63(23.2%)	<0.001
Non-fatal stroke	21(5.3%)	2(1.6%)	19(7.0%)	0.028

## 4 Discussion

The CTO represents one of the most challenging lesion types in coronary intervention, characterized by a pathological process that is prolonged, complex, and systemic in nature (17, 18). Although remarkable advances have been made in catheter-based and imaging techniques in recent years, substantial differences in long-term outcomes among CTO patients persist (19, 20), indicating that anatomical complexity alone is insufficient to comprehensively capture their clinical risk (21). Increasing evidence suggests that systemic nutritional and inflammatory status plays a pivotal role in determining the long-term prognosis of CTO patients (22, 23). In the present study, we found that the combined PNI-GNRI prediction model demonstrated superior performance in assessing the risk of MACCEs within 1 year after PCI in CTO patients. These findings highlight that nutrition-inflammation imbalance may be one of the key mechanisms influencing their prognosis. Consistent with previous studies (24–26), both PNI and GNRI were significantly associated with MACCEs; however, our findings provide additional insight into their complementary roles. PNI, which incorporates serum albumin levels and lymphocyte counts, primarily reflects short-term inflammatory and immune status. This makes it particularly sensitive to acute physiological stress and inflammatory responses that may predispose patients to early cardiovascular events (27). In contrast, GNRI, calculated from serum albumin and the ratio of actual to ideal body weight, serves as a marker of long-term nutritional reserves, reflecting chronic nutritional status and overall physiological resilience (28). The distinction between these indices suggests that PNI and GNRI capture different yet complementary aspects of patient health, explaining why their combined use enhances risk stratification beyond what either measure provides alone. Compared with previous literature, our study emphasizes not only the individual predictive value of each index but also their synergistic utility in clinical practice. CTO lesions are often accompanied by chronic myocardial ischemia, myocardial remodeling, and compensatory development of collateral circulation, with a complex disease course that may persist for months or even years (29, 30). During this process, inflammation and nutritional imbalance are closely interconnected. Hypoalbuminemia is not only a direct manifestation of malnutrition but also a consequence of chronic inflammatory activation (31). Pro-inflammatory cytokines such as IL-6 and TNF-α suppress albumin synthesis, resulting in reduced plasma oncotic pressure and impaired endothelial barrier function, thereby exacerbating edema and hemodynamic instability (32). During inflammation, hepatic protein synthesis, including that of albumin, may be impaired, thereby favoring the production of pro-inflammatory cytokines (33). Malnutrition leads to impaired immune cell function, including dysfunction of macrophages and T lymphocytes. This hampers the effective clearance of cellular debris and inflammatory mediators in the infarcted myocardium, thereby prolonging the inflammatory response (34). After myocardial injury, the innate immune system initiates an inflammatory cascade that activates monocytes, triggers neutrophil-mediated inflammation, and recruits macrophage and lymphocyte subsets to support tissue repair and restore immune balance. These adaptive responses enable the heart to transiently cope with increased stress (35). Immunosuppression also increases susceptibility to infections, adding further systemic burden (36). Prolonged nutritional–immune imbalance compromises vascular repair mechanisms, heightening the risks of intravascular thrombosis and in-stent restenosis. Inflammatory mediators activate signaling pathways such as NF-κB and STAT3, inhibiting endothelial cell proliferation and migration and impairing angiogenesis (37); sustained activation of the IL-6/STAT3 axis suppresses vascular endothelial growth factor (VEGF) signaling, thereby hindering collateral vessel formation and resulting in insufficient revascularization (38). Moreover, the GNRI not only reflects serum albumin levels but also incorporates the ratio of actual to ideal body weight, capturing long-term nutritional depletion and muscle loss (39). Muscle atrophy can result in metabolic and endocrine disturbances as well as impaired muscle contractility, and is often accompanied by hypoalbuminemia, which in turn affects systemic metabolism, immune function, and inflammatory responses (40). In addition, the lack of muscle contraction reduces the secretion of anti-inflammatory myokines (such as IL-10 and IL-15), while visceral fat accumulation promotes macrophage infiltration and increases pro-inflammatory cytokines, including TNF-α and IL-1β, thereby exacerbating vascular endothelial injury (41). Moreover, the loss of protein triggers compensatory hepatic protein synthesis, leading to an imbalance among coagulation, anticoagulation, and fibrinolytic processes (42). Under the combined influence of low protein and low body weight, myocardial tolerance to ischemia–reperfusion injury declines, and mitochondrial dysfunction becomes more pronounced. This further deranges energy metabolism and ultimately limits cardiac functional recovery. Specifically, mitochondrial calcium facilitates the activation of Krebs cycle dehydrogenases, thereby supporting energy metabolic homeostasis and cardiac function. However, this process is disrupted in heart failure, where impaired mitochondrial calcium uptake leads to NADPH oxidation, excessive ROS generation, and ultimately cardiac dysfunction (43). Collectively, these mechanisms demonstrate that nutrition–inflammation imbalance is not merely a marker of systemic status but also directly participates in the entire process of CTO revascularization through cellular signaling pathways, exerting adverse effects from vessel recanalization to postoperative recovery.

From a clinical perspective, a single indicator is often insufficient to capture the prognostic risk of patients. While the PNI primarily reflects immune–inflammatory status, the GNRI emphasizes long-term nutritional status, their combination provides complementary information, improving sensitivity and specificity in risk stratification. Our findings demonstrate that the combined PNI–GNRI model effectively predicts MACCEs, particularly in elderly CTO patients, who are prone to frailty due to muscle loss, endocrine decline, and chronic low-grade inflammation (44). By capturing systemic status across different temporal dimensions, PNI–GNRI can guide precise perioperative risk assessment and inform postoperative rehabilitation and long-term management. In practice, patients could be stratified into low-, intermediate-, and high-risk groups, with tailored nutritional support, anti-inflammatory therapy, and exercise-based rehabilitation. Dynamic monitoring of PNI–GNRI trajectories may further enhance clinical utility: improvements indicate restoration of metabolic and immune balance, while persistent deficits signal the need for intensified interventions (45). This integrated approach highlights the translational potential of PNI–GNRI in advancing individualized, precision care for CTO patients.

Although this study confirmed the clinical value of the combined PNI-GNRI predictive model in patients with CTO, several issues remain unresolved. First, most investigations on the relationship between nutritional inflammation and CTO prognosis have been limited to clinical observations, with insufficient exploration of the underlying molecular mechanisms. Future research should integrate metabolomics, proteomics, and related approaches to comprehensively elucidate how disturbances in nutritional–inflammatory balance influence cardiovascular outcomes through immunometabolic pathways. Second, PNI-GNRI are static indicators and thus cannot capture the dynamic changes during disease progression. The development of dynamic monitoring strategies, potentially incorporating wearable devices and artificial intelligence, may allow for more accurate prediction of MACCEs risk. Third, interventional studies remain lacking. To date, no randomized controlled trials have demonstrated that improving PNI or GNRI can directly reduce the incidence of MACCEs in CTO patients. Large-scale, multicenter, prospective studies are therefore warranted to assess the causal relationships between PNI, GNRI, and clinical outcomes. If validated, these indices may serve not only as prognostic tools but also as therapeutic targets.

### 4.1 Limitation

First, this study was a single-center, retrospective analysis, which inevitably carries the risk of selection bias and may limit the generalizability of the findings. Second, the follow-up period was restricted to only 1 year, making it impossible to assess the long-term prognostic value of PNI-GNRI in patients with CTO. Third, the sample size was relatively modest, with only 395 patients included, which may reduce the statistical power of the study. Future investigations should aim to recruit larger cohorts with extended follow-up durations and adopt multicenter, prospective study designs to further validate our results. Moreover, such studies would be essential to determine the applicability and robustness of PNI-GNRI across diverse populations and clinical settings.

## 5 Conclusion

This study demonstrates that both PNI-GNRI are independent predictors of MACCEs in patients with CTO undergoing PCI. Compared with traditional risk models, the combined PNI–GNRI model exhibits superior sensitivity and specificity for risk stratification, particularly among patients. Comprehensive assessment of nutritional and inflammatory status not only facilitates precise perioperative risk evaluation but also provides a foundation for individualized interventions and long-term recovery planning. Future multicenter, prospective studies are needed to confirm these findings and assess their applicability across diverse patient populations.

## Data Availability

The raw data supporting the conclusions of this article will be made available by the authors, without undue reservation.

## References

[1] Shaya GE Leucker TM Jones SR Martin SS Toth PP. Coronary heart disease risk: low-density lipoprotein and beyond. Trends Cardiovasc Med. (2022) 32:181–94. doi: 10.1016/j.tcm.2021.04.002, PMID: 33872757

[2] Wang W Li J Liu Y Ye P Xu C Yin P . Spatiotemporal trends and ecological determinants of cardiovascular mortality among 2844 counties in mainland China, 2006-2020: a Bayesian modeling study of national mortality registries. BMC Med. (2022) 20:467. doi: 10.1186/s12916-022-02613-9, PMID: 36451190 PMC9714200

[3] Duggan JP Peters AS Trachiotis GD Antevil JL. Epidemiology of coronary artery disease. Surg Clin North Am. (2022) 102:499–516. doi: 10.1016/j.suc.2022.01.007, PMID: 35671770

[4] Bauersachs R Zeymer U Brière JB Marre C Bowrin K Huelsebeck M. Burden of coronary artery disease and peripheral artery disease: a literature review. Cardiovasc Ther. (2019) 2019:1–9. doi: 10.1155/2019/8295054, PMID: 32099582 PMC7024142

[5] Patel VG Brayton KM Tamayo A Mogabgab O Michael TT Lo N . Angiographic success and procedural complications in patients undergoing percutaneous coronary chronic total occlusion interventions: a weighted meta-analysis of 18,061 patients from 65 studies. JACC Cardiovasc Interv. (2013) 6:128–36. doi: 10.1016/j.jcin.2012.10.011, PMID: 23352817

[6] Yang Q Ni S. Prognostic value of malnutrition using geriatric nutritional risk index in patients with coronary chronic total occlusion after percutaneous coronary intervention. Clin Nutr. (2021) 40:4537. doi: 10.1016/j.clnu.2021.05.023, PMID: 34229257

[7] Liang L Zhao X Huang L Tian P Huang B Feng J . Prevalence and prognostic importance of malnutrition, as assessed by four different scoring systems, in elder patients with heart failure. Nutr Metab Cardiovasc Dis. (2023) 33:978–86. doi: 10.1016/j.numecd.2023.01.004, PMID: 36710105

[8] Wu L Wang W Gui Y Yan Q Peng G Zhang X . Nutritional status as a risk factor for new-onset atrial fibrillation in acute myocardial infarction. Clin Interv Aging. (2023) 18:29–40. doi: 10.2147/CIA.S387602, PMID: 36644454 PMC9838126

[9] Fernández-Lázaro D Seco-Calvo J. Nutrition, nutritional status and functionality. Nutrients. (2023) 15:1944. doi: 10.3390/nu15081944 37111162 PMC10142726

[10] Jarosz I Gorecki K Kalisz G Popiolek-Kalisz J. Nutritional status assessment tools in cardiovascular patients. Nutrients. (2025) 17:2703. doi: 10.3390/nu17162703, PMID: 40871731 PMC12389674

[11] Zhang S Wang H Chen S Cai S Zhou S Wang C . Prognostic nutritional index and prognosis of patients with coronary artery disease: a systematic review and meta-analysis. Front Nutr. (2023) 10:1114053. doi: 10.3389/fnut.2023.1114053, PMID: 37006923 PMC10061069

[12] Wada H Dohi T Miyauchi K Jun S Endo H Doi S . Relationship between the prognostic nutritional index and long-term clinical outcomes in patients with stable coronary artery disease. J Cardiol. (2018) 72:155–61. doi: 10.1016/j.jjcc.2018.01.012, PMID: 29496336

[13] Koyuncu I Koyun E. Relationship between HALP and PNI score with 1-month mortality after CABG. Front Nutr. (2024) 11:1489301. doi: 10.3389/fnut.2024.1489301, PMID: 39555199 PMC11563828

[14] Bouillanne O Morineau G Dupont C Coulombel I Vincent JP Nicolis I . Geriatric nutritional risk index: a new index for evaluating at-risk elderly medical patients. Am J Clin Nutr. (2005) 82:777–83. doi: 10.1093/ajcn/82.4.777, PMID: 16210706

[15] Fan Y He L Zhou Y Man C. Predictive value of geriatric nutritional risk index in patients with coronary artery disease: a meta-analysis. Front Nutr. (2021) 8:736884. doi: 10.3389/fnut.2021.736884, PMID: 34660665 PMC8511313

[16] Okadome K Baba Y Yagi T Kiyozumi Y Ishimoto T Iwatsuki M . Prognostic nutritional index, tumor-infiltrating lymphocytes, and prognosis in patients with esophageal cancer. Ann Surg. (2020) 271:693–700. doi: 10.1097/SLA.0000000000002985, PMID: 30308614

[17] Guelker JE Kinoshita Y Weber-Albers J Bufe A Blockhaus C Mashayekhi K. Validation of the newly introduced CASTLE score for predicting successful CTO recanalization. IJC Heart Vasc. (2022) 38:100942. doi: 10.1016/j.ijcha.2021.100942, PMID: 35079620 PMC8777279

[18] Melotti E Belmonte M Gigante C Mallia V Mushtaq S Conte E . The role of multimodality imaging for percutaneous coronary intervention in patients with chronic total occlusions. Front Cardiovasc Med. (2022) 9:823091. doi: 10.3389/fcvm.2022.823091, PMID: 35586657 PMC9108201

[19] Kalnins A Strele I Lejnieks A. Comparison among different scoring systems in predicting procedural success and long-term outcomes after percutaneous coronary intervention in patients with chronic total coronary artery occlusions. Medicina (Kaunas). (2019) 55:494. doi: 10.3390/medicina55080494 31426403 PMC6724017

[20] Brilakis ES Mashayekhi K Tsuchikane E Abi Rafeh N Alaswad K Araya M . Guiding principles for chronic total occlusion percutaneous coronary intervention. Circulation. (2019) 140:420–33. doi: 10.1161/CIRCULATIONAHA.119.039797, PMID: 31356129

[21] Schumacher SP Stuijfzand WJ Opolski MP van Rossum AC Nap A Knaapen P. Percutaneous coronary intervention of chronic Total occlusions: when and how to treat. Cardiovasc Revasc Med. (2019) 20:513–22. doi: 10.1016/j.carrev.2018.07.025, PMID: 30093292

[22] Dündar C Tanalp AC Yağmur A Karaaslan MB. 2025 The impact of the Naples prognostic score in long-term outcomes after percutaneous coronary intervention for chronic total occlusions. Coron Artery Dis, 36:422–427. 39912314 10.1097/MCA.0000000000001512

[23] Li C Zhang F Shen Y Xu R Chen Z Dai Y . Impact of neutrophil to lymphocyte ratio (NLR) index and its periprocedural change (NLR(Δ)) for percutaneous coronary intervention in patients with chronic total occlusion. Angiology. (2017) 68:640–6. doi: 10.1177/0003319716649112 27207843

[24] Nakashima M Akagi S Ejiri K Nakamura K Ito H. Impact of malnutrition on prognosis in patients with pulmonary arterial hypertension. Pulm Circ. (2023) 13:e12286. doi: 10.1002/pul2.12286, PMID: 37705961 PMC10496044

[25] Ishiwata S Yatsu S Kasai T Sato A Matsumoto H Shitara J . Prognostic effect of a novel simply calculated nutritional index in acute decompensated heart failure. Nutrients. (2020) 12:3311. doi: 10.3390/nu12113311 33137941 PMC7694067

[26] Wang Q Ye Z Rao J Zhao W Liu J Chen Y . Assessing the predictive power of nutritional indices on all-cause and cardiovascular mortality in hemodialysis patients: a longitudinal study. Ren Fail. (2024) 46:2431140. doi: 10.1080/0886022X.2024.2431140, PMID: 39608361 PMC11610283

[27] Zhao J Liu K Li S Gao Y Zhao L Liu H . Prognostic nutritional index predicts clinical outcomes in patients with cerebral venous sinus thrombosis. BMC Neurol. (2021) 21:404. doi: 10.1186/s12883-021-02436-w, PMID: 34674659 PMC8529735

[28] Er B Mızrak B Aydemir A Binay S Doğu C Kazancı D . Association of nutritional status, frailty, and rectus femoris muscle thickness measured by ultrasound and weaning in critically ill elderly patients. Tuberk Toraks. (2023) 71:1–6. doi: 10.5578/tt.20239901, PMID: 36912403 PMC10795233

[29] Chen Y Zheng X Jin H Deng S Ren D Greiser A . Role of myocardial extracellular volume fraction measured with magnetic resonance imaging in the prediction of left ventricular functional outcome after revascularization of chronic total occlusion of coronary arteries. Korean J Radiol. (2019) 20:83–93. doi: 10.3348/kjr.2018.0069, PMID: 30627024 PMC6315067

[30] Xiao K Xv Z Xv Y Wang J Xiao L Kang Z . H-type hypertension is a risk factor for chronic total coronary artery occlusion: a cross-sectional study from Southwest China. BMC Cardiovasc Disord. (2023) 23:301. doi: 10.1186/s12872-023-03345-1, PMID: 37328790 PMC10273712

[31] Ahmed MS Jadhav AB Hassan A Meng QH. Acute phase reactants as novel predictors of cardiovascular disease. ISRN Inflamm. (2012) 2012:953461. doi: 10.5402/2012/953461, PMID: 24049653 PMC3767354

[32] Lin W Fu C Miao J Hong W Chen X Yan S . Association between the serum albumin-creatinine ratio and 28-day intensive care unit mortality among patients with sepsis: a multicenter retrospective cohort study. Front Med. (2024) 11:1484370. doi: 10.3389/fmed.2024.1484370, PMID: 39564496 PMC11573561

[33] Sacchetti F Caprino P Potenza AE Pastena D Presacco S Sofo L. Early and late outcomes of a series of 255 patients with Crohn's disease who underwent resection: 10 years of experience at a single referral center. Update Surg. (2022) 74:1657–64. doi: 10.1007/s13304-022-01322-5, PMID: 35841530 PMC9481492

[34] Zheng X Zheng X Zhang C Liu M. Geriatric nutritional risk index as a predictor of 30-day and 365-day mortality in patients with acute myocardial infarction: a retrospective cohort study using the MIMIC-IV database. Front Nutr. (2025) 12:1544382. doi: 10.3389/fnut.2025.1544382, PMID: 39973920 PMC11835668

[35] Zhao Y Tan M Yin Y Zhang J Song Y Li H . Comprehensive macro and micro views on immune cells in ischemic heart disease. Cell Prolif. (2024) 57:e13725. doi: 10.1111/cpr.13725, PMID: 39087342 PMC11628753

[36] Greenwood H Patel J Mahida R Wang Q Parekh D Dancer RC . Simvastatin to modify neutrophil function in older patients with septic pneumonia (SNOOPI): study protocol for a randomised placebo-controlled trial. Trials. (2014) 15:332. doi: 10.1186/1745-6215-15-332, PMID: 25146127 PMC4247744

[37] Xiong J Zhang D Wu J Yang P Shi D Zhou X . Association between neutrophil-lymphocyte ratio and female breast cancer: an observational study from NHANES 2001-2018 with external validation. Front Oncol. (2025) 15:1564238. doi: 10.3389/fonc.2025.1564238, PMID: 40697377 PMC12280361

[38] Dai X Faber JE. Endothelial nitric oxide synthase deficiency causes collateral vessel rarefaction and impairs activation of a cell cycle gene network during arteriogenesis. Circ Res. (2010) 106:1870–81. doi: 10.1161/CIRCRESAHA.109.212746, PMID: 20431061 PMC3401938

[39] Xie Y Xiong Y Sun M Zhao Y Wu M. Research trends in nutritional interventions for stroke: a bibliometric analysis and literature review. Front Nutr. (2024) 11:1489222. doi: 10.3389/fnut.2024.1489222, PMID: 39483787 PMC11526124

[40] Fang WN Wu HX Wu ZP Fei ZD Zhao D Chen F . A scoring system based on inflammatory and nutritional indicators to predict the long-term survival of patients with non-metastatic nasopharyngeal carcinoma. Sci Rep. (2024) 14:20229. doi: 10.1038/s41598-024-71360-z, PMID: 39215059 PMC11364744

[41] Huang X Hu L Li J Meng C Xia X Liu Y. Dietary intake of live microbes mitigates the mortality risk associated with sedentary behavior in US hypertensive individuals. Sci Rep. (2025) 15:16483. doi: 10.1038/s41598-025-01122-y, PMID: 40355534 PMC12069680

[42] Li Y Zhang M Xue M Wei M He J Dong C. A case report of cerebral venous sinus thrombosis presenting with rapidly progressive dementia. Front Med. (2022) 9:985361. doi: 10.3389/fmed.2022.985361, PMID: 36091714 PMC9452803

[43] Li A Zheng N Ding X. Mitochondrial abnormalities: a hub in metabolic syndrome-related cardiac dysfunction caused by oxidative stress. Heart Fail Rev. (2022) 27:1387–94. doi: 10.1007/s10741-021-10109-6, PMID: 33950478 PMC9197868

[44] Naito A Nagatomo Y Kawai A Yukino-Iwashita M Nakazawa R Taruoka A . The safety and efficacy of sodium-glucose cotransporter-2 inhibitors for patients with sarcopenia or frailty: double edged sword? J Pers Med. (2024) 14:141. doi: 10.3390/jpm14020141, PMID: 38392575 PMC10890336

[45] Vente JP Soeters PB von Meyenfeldt MF Rouflart MM van der Linden CJ Gouma DJ. Prospective randomized double-blind trial of branched chain amino acid enriched versus standard parenteral nutrition solutions in traumatized and septic patients. World J Surg. (1991) 15:128–132, 133. doi: 10.1007/BF01658984 1899735

